# Autophagy Modulation in Lymphocytes From COVID-19 Patients: New Therapeutic Target in SARS-COV-2 Infection

**DOI:** 10.3389/fphar.2020.569849

**Published:** 2020-11-19

**Authors:** Marta Vomero, Cristiana Barbati, Tania Colasanti, Alessandra Ida Celia, Mariangela Speziali, Federica Maria Ucci, Claudia Ciancarella, Fabrizio Conti, Cristiano Alessandri

**Affiliations:** Rheumatology Unit, Department of Clinical, Internal, Anesthesiological and Cardiovascular Sciences, Sapienza University of Rome, Rome, Italy

**Keywords:** SARS-CoV-2, CoViD-19, autophagy, lymphocytes, cytokines

## Abstract

Severe Acute Respiratory Syndrome Coronavirus 2 (SARS-CoV-2) is the novel coronavirus, causing coronavirus disease 2019 (COVID-19). During virus infection, several pro-inflammatory cytokines are produced, leading to the “cytokine storm.” Among these, interleukin (IL)-6, tumor necrosis factor‐α (TNF‐α), and IL-1β seem to have a central role in the progression and exacerbation of the disease, leading to the recruitment of immune cells to infection sites. Autophagy is an evolutionarily conserved lysosomal degradation pathway involved in different aspects of lymphocytes functionality. The involvement of IL-6, TNF‐α, and IL-1β in autophagy modulation has recently been demonstrated. Moreover, preliminary studies showed that SARS-CoV-2 could infect lymphocytes, playing a role in the modulation of autophagy. Several anti-rheumatic drugs, now proposed for the treatment of COVID-19, could modulate autophagy in lymphocytes, highlighting the therapeutic potential of targeting autophagy in SARS-CoV-2 infection.

## Introduction

The outbreak of coronavirus disease 2019 (COVID-19), caused by the 2019 novel Severe acute respiratory syndrome Coronavirus 2 (SARS-CoV-2), started in Wuhan, Hubei, China, in December 2019. World Health Organization (WHO) declared COVID-19 a pandemic by March 2020 (Wu et al., 2020). SARS-CoV-2 is extremely contagious; the progression to acute respiratory distress syndrome (ARDS) and multi-organ failure is often rapid and dramatic. At the time of writing this report, COVID-19 global cases are 26, 994, 442, with 880,994 deaths (data as received by WHO from national authorities by September 7, 2020; https://covid19.who.int/). During ARDS, active pulmonary epithelial cells, together with infiltrating adaptive and innate immune cells, are responsible for the aberrant production of pro-inflammatory molecules (cytokine storm), contributing to an excessive recruitment of inflammatory cells and to the local release of proteases and oxidants that are involved in pulmonary manifestations of the disease (Channappanavar et al., 2017). In this scenario, different cytokines, including IL-6, TNF-α, and IL-1β, support the disease’s inflammatory-associated events (Chen et al., 2020). Cytokine release at a local or systemic level characterizes the more severe phase of COVID-19. It may compromise the immune response against viral infection, often leading to organ damage and even death (Girija et al., 2020). Besides, innate immune cells are critical populations candidates to produce cytokines responding to inflammation and infection in the body, comprising endothelial cells, adipocytes, and mast cells. During SARS-CoV-2 infection, adipocytes, produce IL-6, TNF-α, and IL-1β, contributing to the worsening of the host response to pathogens (Michalakis et al., 2020).

Autophagy is an evolutionarily conserved lysosomal degradation pathway involving the segregation of cytoplasmic intracellular material, including proteins and organelles, in a structure with a double membrane, called autophagosome (Ortona et al., 2014; Pierdominici et al., 2014). Recently, autophagy has emerged as a fundamental process in regulating T and B lymphocytes’ maturation and survival. Also, defects in the autophagy process have been associated with the pathogenesis and progression of several immuno-mediated diseases.

Our research group demonstrated different mechanisms by which autophagy participates in the pathogenesis of autoimmune disorders. We found increased autophagy levels in naive CD4^+^ T lymphocytes of systemic lupus erythematosus (SLE) patients. We identified the *α*-synuclein as a new autophagy marker in T lymphocytes (Alessandri et al., 2012; Colasanti et al., 2014). We described that not only TNFα induced autophagy in peripheral blood mononuclear cells from rheumatoid arthritis (RA) patients, but also that the balance between autophagy and apoptosis was involved in response to therapy in RA patients treated with TNF inhibitors (Vomero et al., 2019).

A recent study showed, in a model of cardiac fibrosis, how IL-6 can be involved in autophagy regulation by activating Signal Transducer and Activator of Transcription 3 (STAT3) and it can also cause autophagy up-regulation in vascular smooth muscle cells (An et al., 2017; Billah et al., 2020). Furthermore, it has been demonstrated the role of autophagy in IL-1β production (Joosten et al., 2013). Moreover, the capacity of IL-1β in inducing autophagy and apoptosis in human degenerative nucleus pulposus cells has been described (Shen J et al., 2017).

T cells susceptibility to SARS-CoV-2 infection has been recently demonstrated. Wang et al. showed that SARS-CoV-2 could infect T cells via a receptor-dependent spike protein-mediated membrane fusion (Wang X. et al., 2020). It can be hypothesized that the virus spike protein is able to mediate the strong infectious potential, even on cells presenting low levels of ACE2 receptor (such as T lymphocytes). To understand the reason for the elevated transmission rate of SARS-CoV-2, the authors hypothesized that other receptors, such as CD147, may be involved in SARS-CoV-2 entrance into lymphocytes (Wang X. et al., 2020).

During viral infection, the autophagy machinery could have a double face. On the one hand, the autophagy breakdown of cytoplasmic viral components or entire virions, also known as virophage, could oppose virus entry and replication (Galluzzi et al., 2017). However, many viruses can escape these inhibitory functions and benefit autophagy components for their entry, replication, and exit (Jackson, 2015).

In this regard, the well-studied mouse hepatitis virus (MHV), used as a not dangerous model to study CoV infection, induces the formation of double-membrane vesicles (DMVs), similarly to the autophagosome. As the complexes for virus replication at DMVs level colocalize with the major autophagy marker LC3 and MHV replication seems to be impaired in ATG5 knockout embryonic stem cells, several studies conclude that autophagy could be involved in the development of DMVs, and in the replication of MHV (Yang et al., 2020).

The importance of endosome-lysosome emerged from a study in which the infection of CoVs infectious bronchitis virus and Porcine Epidemic Diarrhea Virus was followed by an accumulation of the viruses in the lysosomes of cells. Therefore, several studies considered the endocytic pathway as a target of anti-viral therapies. SARS-CoV-2 has been widely demonstrated to take advantage of the endocytic pathway for viral entry into various host cells (Yang et al., 2020).

## Role of Autophagy Inhibition by Chloroquine/hydroxychloroquine in SARS-CoV-2 Infection

Chloroquine (CQ) and hydroxychloroquine (HCQ) are antimalarial drugs used as disease-modifying antirheumatic drugs (DMARDs), empirically introduced for the treatment of various rheumatological diseases. Several studies of antimalarial drugs in SLE, with a specific reference to HCQ, defined that this molecule has an immunomodulatory and anti-inflammatory property.

CQ and HCQ have a well-established role in compromising lysosomal activity and, consequently, in the autophagy mechanism. (Mauthe et al., 2018) CQ can accumulate into lysosome where, by increasing the pH of this organelle, it interferes with the formation of autophagolysosome (Schrezenmeier et al., 2020). The role of lysosomes is to recycle cellular substrates, but they are also involved in antigen processing and presentation, thus stimulating the immune response. These mechanisms explain the antiviral activity of CQ, which was demonstrated *in vitro* for the first time in 1969 (Inglot, 1969; Ballabio et al., 2019).

During the last years, various of new viral infections have emerged without a well-defined therapy. Therefore, some researches are focused on repurposing available medications for the management of these infections. *In vitro*, *in vivo*, and human studies have been performed on anti-malarial drugs, including HCQ and CQ, and some encouraging observations have been reported (Savarino et al., 2003). Most available studies are focused on HIV and showed acceptable efficacy, especially as an adjuvant treatment besides routine HAART (Naghipour et al., 2020). Despite this, human studies are lacking for some infections like ZIKA, EBOLA, SARS-CoV and MERS-CoV.

HCQ was initially proposed for the treatment of COVID-19 patients. However, due to safety issues, the WHO has recommended stopping the use of HCQ. Literature reports contrasting findings even though, to date, no RCT has shown an absolute advantage in preventing or improving the major outcomes in COVID-19 patients. An observational study conducted by Geleris et al., on 1,376 patients who had been hospitalized with COVID-19 failed to detect a potential benefit of HCQ and colchicine (Geleris et al., 2020). A multicenter, randomized, open-label, controlled trial showed that among patients hospitalized with mild-to-moderate COVID-19, the use of HCQ, alone or with azithromycin, did not improve patients health status at 15 days as compared with standard care (Cavalcanti et al., 2020). A clinical trial testing two doses of CQ in patients with COVID-19 planned to include 440 patients. Still, it was halted after 81 patients had been enrolled because of excessive QTc prolongation and an indication of higher mortality in the high-dose group than in the low-dose group (Borba et al., 2020). In a letter to the editor recently published in the International Journal of Infectious Diseases, it has been shown a reduction of mortality in an Italian cohort of COVID-19 patients treated with a combination of HCQ plus azithromycin compared to the control group (d'Arminio Monforte et al., 2020). In a previous paper, Arshad and colleagues studied hospital mortality in a cohort of patients treated with HCQ, demonstrating that the administration of this drug alone and in combination with azithromycin was associated with a lower risk of death in hospitalized patients affected by COVID-19 (Arshad, 2020).

Although the efficacy of HCQ in COVID-19 treatment is still under investigation, several experimental evidences suggest that this drug could have a role in different aspects of COVID-19, including reducing inflammation and virus elimination via autophagy inhibition.

It has been demonstrated that CQ and HCQ were able to reduce the release of some pro-inflammatory cytokines, such as TNF-α and IL-6, from circulating mononuclear cells (van den Borne, 1997). Although additional evidences are needed, this study suggests a possible involvement of HCQ in the reduction of symptoms associated with cytokine storm in patients with COVID-19.

Chloroquine prevents terminal glycosylation of the ACE2 receptor for cell entry, recognized by SARS-CoV and SARS-CoV-2 (Devaux et al., 2020). Moreover, it was shown that in cells treated with CQ, the viral receptor ACE2 was engulfed into perinuclear vacuoles, proposing that these lysosomotropic agents may interfere with the function of ACE2 (Wang et al., 2008). Additional mechanism by which CQ exerts its antiviral activity consists in the change of lysosomes pH, leading to inhibition of the cathepsins. These enzymes play a crucial role in developing autophagosomes cleaving the SARS-CoV-2 spike protein and blocking the viral adhesion to the human host receptors (Wang M. et al., 2020). The antiviral activity of HCQ was recently investigated also by Yao and colleagues; these authors confirmed the capacity of HCQ of reducing SARS-CoV-2 replication in Vero cells *in vitro* infected by the virus (Yao, et al., 2020).

Human coronaviruses also depend on sialic-acid-containing glycoproteins and gangliosides that act as primary adhesion molecules along the respiratory tract (Matrosovich et al., 2015). Furthermore, CQ can bind the quinone reductase-2, an essential factor required for synthesizing sialic acid, generally used as the receptor portion by the SARS-CoV-2 (Singh et al., 2020).

**FIGURE 1:**
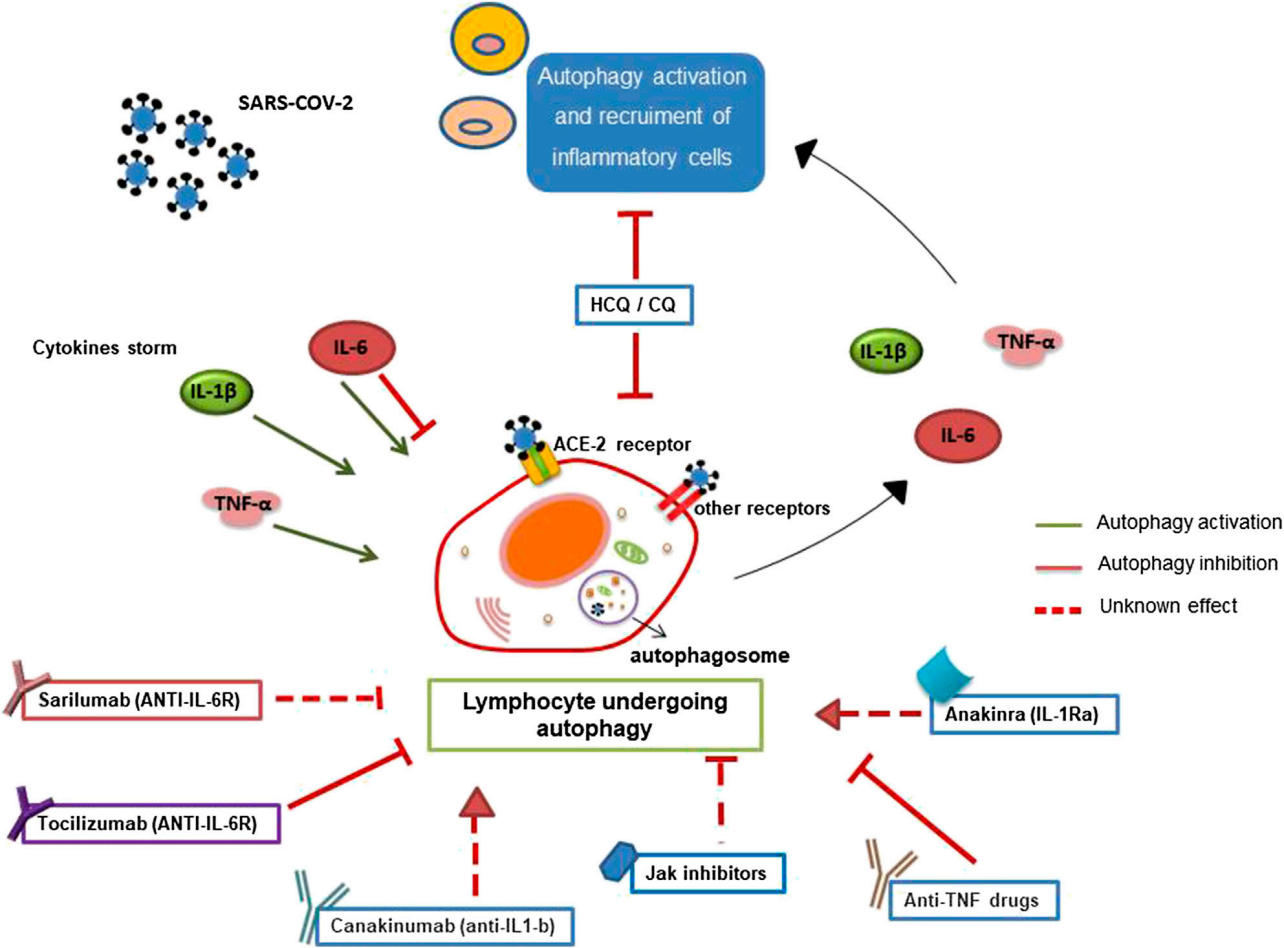
Involvement of autophagy in lymphocyte-mediated inflammation during SARS-CoV-2 infection. Autophagy has a crucial role in the survival, activation, and lymphocyte function. Pro-inflammatory cytokines, secreted during SARS-CoV-2 infection, could influence autophagy contributing to cytokine storm and inflammation. Furthermore, SARS-CoV-2 binding the ACE-2 lymphocyte receptor or the other putative receptors (such as CD147) could play a role in the modulation of autophagy. Several anti-rheumatic drugs, now proposed for the treatment of COVID-19, could target autophagy.

Since CQ/HCQ was demonstrated to inhibit SARS-CoV-2 infection *in vitro*, we suppose that these drugs can exert their antiviral role, inhibiting autophagy involved in SARS-CoV-2 entry replication (Figure 1).

## Autophagy Modulation by Glucocorticoids in SARS-CoV-2 Infection

Glucocorticoids are a class of steroid hormones widely used to treat inflammatory and autoimmune diseases for their immunosuppressive action. The advantage of including glucocorticoids in the therapy of patients affected by COVID-19 is under investigation, and up to now studies are inconclusive (Stockman et al., 2006). Increasing evidence showed that clinical management of glucocorticoids should consider the two different pathophysiological phases of SARS-CoV-2 infection. In fact, at the beginning of the infection, glucocorticoids could downregulate innate immune response against the virus and favor viral entry into cells; however, the use of glucocorticoids, in particular of methylprednisolone, may be beneficial in condition of lung damage and cytokine storm due to their capacity of reducing fibrosis and inflammation (Veronese et al., 2020).

Although the mechanism is yet to be elucidated, experiments in T cell lymphoma line demonstrated that dexamethasone could induce both apoptosis and autophagy (Swerdlow et al., 2008). The authors demonstrated that in patients treated with glucocorticoids, autophagy may have a role in the prolonged survival of cancer cells and that autophagy inhibition could be beneficial. Cells in which autophagy was pharmacologically inhibited showed increased levels of apoptosis mediated by glucocorticoid. The same authors found that lymphocytes treated *in vitro* with glucocorticoid upregulated dexamethasone-induced gene 2 (Dig2), a negative regulator of mTOR protein inhibitor autophagy (Molitoris et al., 2011).

Moreover, since it is well known that glucocorticoids inhibit the uptake of glucose in lymphocytes *in vitro* (Kattwinkel et al., 1966) and lymphocytes activate autophagy in response to serum deprivation, it could be possible that the induction of autophagy mediated by glucocorticoids may represent a response to stress condition. In support of this hypothesis, Totino and colleagues found that peripheral lymphocytes were treated with dexamethasone undergoing a particular cell death process similar to autophagy and characterized by a massive vacuolization of the cytoplasm (Totino et al., 2006). Interestingly, in a most recent article, the role of autophagy in dexamethasone-resistant leukemia cells has been proposed (Jiang et al., 2015). The combination of dexamethasone and the autophagy-inhibitor CQ caused not only apoptosis but also a reduction of proliferation in lymphoid malignant cell lines.

Taking together, these results confirm the importance of the interplay between autophagy and apoptosis in lymphocytes activation and suggest that a similar scenario might occur in patients affected by COVID-19. Although studies on the autophagic behavior of lymphocytes from COVID-19 patients are mandatory, in SARS-CoV-2 infection, autophagy induced by glucocorticoids might represent a mechanism of lymphocytes survival, and inhibition of autophagy, for example, by antimalarial drug HCQ, might reactivate apoptosis of lymphocytes suppressing thus the excessive inflammatory response.

## Autophagy Modulation by Targeting Cytokines in SARS-CoV-2 Infection

### IL-6

In SARS-CoV-2–infected subjects, IL-6 rises during disease and declines during recovery. Moreover, hospitalized patients in intensive care had high levels of IL-6 (Diao et al., 2020). IL-6 is a pleiotropic cytokine involved in systemic immune defense against external pathogens inducing a defensive response mediated by a wide range of cells, including B and T lymphocytes, monocytes/macrophages and neutrophils (Dayer et al., 2010). It remains elusive whether IL-6 and autophagy are linked. Recent studies reported that IL-6 might have both inhibitory and stimulating effects on autophagy (Kimura et al., 2010). It has been shown that the upregulation of autophagy by IL-6 protects pancreatic beta cells from apoptosis (Linnemann et al., 2017). On the contrary, Pinto and colleagues showed that the inhibition of autophagy proteins in the liver, induced by the acute exhaustive physical exercise, was attenuated in IL-6 knock out mice (Pinto et al., 2020). However, the role of IL-6 in autophagy modulation is ambiguous. It is well known that IL-6 activates STAT3, by interaction with Janus-activated kinases (Jak), and recently several studies indicated an involvement of STAT3 in autophagy regulation (Wang Y. et al., 2013). In this regard, IL-6 promotes phosphorylation of STAT3 (p-STAT3), induces its mitochondrial translocation, and activates autophagy (Kang et al., 2012). On the contrary, other data showed that p-STAT3 inhibits the autophagic pathway. Yamada et al. demonstrated that fyntyrosine-kinase promotes the activation of STAT3 by reducing the VPS34 level, which inhibits autophagy in skeletal muscle fiber (Yamada et al., 2012). Furthermore, Yokoyama and colleagues showed a different mechanism by which autophagy was induced through inhibition of STAT3 (Yokoyama et al., 2007). The study of Kimura et al. confirmed these results, showing that the inhibition of autophagic cell death by activation of STAT3 signaling via IL‐6, attenuated arsenite‐induced renal injury (Kimura et al., 2010). Besides, the cytoplasmic non-phosphorylated form of STAT3 represses autophagy by inhibiting its enzymatic activity of PKR, and consequently, the phosphorylation of eIF2α, responsible for autophagy induction (Shen S et al., 2012). In summary, STAT3 is strongly involved in the autophagy modulation. It is also known that STAT3 regulates lymphocytes function and differentiation (Kane et al., 2014). No data regarding the role of IL-6 on autophagy modulation in lymphocytes are present in the literature. However, our preliminary results observed in RA patients’ lymphocytes showed that autophagy is inhibited after *in vitro* treatment with an anti-Jak inhibitor. Also, a reduction of autophagy-related markers was found in peripheral blood immune cells isolated from RA patients treated with this drug (unpublished data). These results suggest a direct role of IL-6 in modulating lymphocytes autophagy.

The prominent role of IL-6 in SARS-CoV-2 infection has led to IL-6 inhibitors’ administration in the clinical practice. Different clinical trials on the use of tocilizumab and sarilumab, two IL-6 receptor-inhibiting monoclonal antibodies, in patients with COVID-19 are ongoing. In this regard, in glioblastoma cells undergoing hypoxia, stress stimuli, autophagy was activated, and the expression of IL-6 resulted upregulated. The numbers of autophagosomes in IL-6-treated cells were increased, suggesting that this cytokine can modulate autophagy. In addition, a blockade of both endogenous and exogenous IL-6 by the treatment with tocilizumab was able to repress autophagy in glioblastoma cells. The authors suggest that tocilizumab promotes apoptosis on glioma cells by antagonizing hypoxia-induced autophagy (Xue et al., 2016).

Similarly, Jak inhibitors, such as tofacitinib and baricitinib, have been proposed as part of the treatment of COVID-19.

It can be reasonable to hypothesize that these drugs could improve the therapeutic response in COVID-19 patients also by modulating lymphocytes autophagy (Figure 1).

## TNF-α

Similarities in serum cytokines panel of COVID-19-infected subjects and patients affected by autoimmune diseases suggest that some immune-mediated mechanisms might be familiar to both. A recent paper on COVID-19 positive patients immunological characteristics showed higher levels of TNF-α in severe cases than in moderate cases (Chen et al., 2020). TNF-α is secreted by activated monocytes and macrophages, but also by a lot of other cell types, including lymphocytes. TNF-α is produced as a transmembrane protein, and then, thanks to the proteolytic cleavage by the enzyme TACE, it is released in its soluble homotrimeric form (Kruglov et al., 2008). TNF-α can activate both survival and cell death mechanisms by modulating cellular signaling. It can significantly stimulate apoptosis by activating the cell death receptor and the expression of anti-apoptotic and pro-survival genes by NF-kB (Yamasaki et al., 2001). It also promotes mechanisms such as monocytes activation, production and release of other pro-inflammatory molecules, the release of metalloproteases, and lymphocytes recruitment to inflamed tissues, including lung. Consequently, clinical trials based on TNF inhibitors have been recently proposed in patients with COVID-19 (Feldmann et al., 2020). It was shown that TNF-α induced autophagy in both non-immune and immune cell types. We previously demonstrated that TNF-α activated autophagy in peripheral lymphocytes from RA patients. The treatment with drugs that block this cytokine, such as etanercept, reduced *in vitro* the autophagy marker LC3-II (Vomero et al., 2019). Only patients that responded to anti-TNF therapy showed a significant reduction in autophagy levels. In addition, we showed that TNF-α carried on microparticles purified from RA patients was able to upregulate autophagy on endothelial cells. *In vitro* and *ex vivo* treatment with etanercept blocked this effect (Barbati et al., 2018).

Autophagy plays an important role in the immune-mediated response of the lung to infection. By regulating the clearance of damaged proteins and/or organelles and ROS production, in average condition, autophagy is responsible for tissue homeostasis and prevention of spontaneous pulmonary inflammation. During lung infection, autophagy actively participates in host defense, not only stimulating the degradation of pathogens into autophagolysosomes, but also activating a specific immune response. It has been shown that pulmonary lymphocytes activate autophagy to sustain their activation, functions, and production of cytokines (Racanelli et al., 2018). However, persistent or deregulated autophagy can prompt an excessive immune stimulation contributing to chronic lung diseases.

In lungs, autophagy is involved in activating innate immune response against *Mycobacterium tuberculosis*, the agent that causes *tuberculosis*. In macrophages, autophagy started by TNF-α is involved in the killing of *Mycobacterium tuberculosis*. Patients treated with anti-TNF drugs are subjected to mycobacteria reactivation due to autophagy downregulation (Harris et al., 2010).

As shown in Figure 1, cytokines, including TNF-α, induce, through a feedback mechanism, autophagy, causing further recruitment and activation of lymphocytes, and contributing to the excess of inflammation typical of SARS-CoV-2 infection. Although experimental evidence is needed, these observations suggest a possible role of lymphocyte autophagy in TNF-mediated inflammatory excess in patients with COVID-19.

## IL-1β

A small number of patients affected by COVID-19 develop mild or highly acute respiratory syndrome characterized by pro-inflammatory cytokines, including IL-1β. SARS-CoV-2 binds Toll-Like Receptors (TLRs), causing the release of pro-IL-1β and inflammasome activation. Inside the inflammasome, the activated caspase-1 cleaves pro-IL-1β, producing the active form of mature IL-1β, a mediator of lung inflammation, fever, and fibrosis.

It has been recently shown that the inhibition of pro-inflammatory IL-1 family members can represent a therapeutic option in many inflammatory diseases, including viral infections (Conti et al., 2020).

Anakinra, a recombinant form of IL-1Ra, was one of the first biological agents approved for RA treatment in Europe in March 2002. Since then, anakinra obtained authorization also for some autoinflammatory syndromes, such as adult-onset Still disease (AOSD). Moreover, canakinumab, a novel human monoclonal antibody targeting IL-1β, is now approved for cryopyrinopathies, AOSD, and gout.

Recently, IL-1β was demonstrated to activate the formation of autophagosomes, and it seems to be involved in the induction of autophagy as part of a negative feedback loop that leads to the reduction of the inflammatory response and the activation of anti-microbial defense mediated by the action of cytokines (Zang et al., 2015).

Little is known about the effect of inhibition of IL-1β on autophagy. Macrophages from patients with chronic granulomatous disease (CGD) displayed a deficit in autophagy activation, and the inhibition of IL-1 by the receptor antagonist anakinra restored autophagy in the CGD mice model (de Luca, 2014.) Furthermore, in the study of Torene et al., canakinumab is described to be involved in the transcription of autophagy genes in some inflammatory responses; however, the number of autophagy- and inflammasome-related genes that were transcriptionally regulated by canakinumab was not sufficient to draw any conclusions about their pathogenetic role (Torene et al., 2017). Consequently, more studies are needed to clarify this aspect.

It is well known a direct effect of IL-1β on lymphocytes activation; in particular, IL-1β is involved in the proliferation and maturation of thymocytes and naïve T cells (Gotlieb et al., 1991), and consequently in the expansion and differentiation of antigen-specific naïve and memory CD4^+^ T cells. Ben-Sasson and colleagues also studied the involvement of IL-1β on maturation of Th1 and Th2 cells (Ben-Sasson et al., 2009). In addition, recent evidence revealed the role of autophagy in regulating inflammation mediated by the inflammasome, a multiprotein complex involved in the secretion of pro-inflammatory cytokines, such as IL-1β, via caspase-1 activation. It has been demonstrated that the inhibition of autophagy led to inflammasome activation and IL-1β release (Nakahira et al., 2011). Kanayama and coauthors demonstrated that in pulmonary myeloid cells autophagy prevents an excessive inflammatory response by downregulating the activation of inflammasome complex and IL-1 *β* production (Kanayama et al., 2015).

Thus, we can hypothesize that autophagy might be a potential target of IL-1R-antagonist/IL-1β inhibitors in lymphocytes. The modulation of autophagy may have a role in the anti-inflammatory effects of these drugs in patients affected by COVID-19 (Figure 1).

## Conclusion

Up to now, autophagy was poorly considered and explored in COVID-19. Since autophagy seems to be involved in both lymphocytes activation and SARS-CoV-2 cell entry and replication, in this perspective article we described lymphocytes autophagy as a possible player in COVID-19. During SARS-CoV-2 infection, cytokine release driven by the virus and by an excessive immune response could deregulate autophagy, contributing to the progression of the disease. Although anti-rheumatic drugs, now proposed for COVID-19 treatment, are able to affect several biological mechanisms, we speculate that they could also modulate autophagy in lymphocytes leading to a reduction of inflammation in patients infected by SARS-CoV-2. The impact of drugs targeting autophagy represents an emerging topic, worth to be considered as a new therapeutic strategy in the context of COVID-19.

## Author Contributions

All authors have made a substantial, direct and intellectual contribution to the work, and approved the final manuscript.

## Conflict of Interest

The authors declare that the research was conducted in the absence of any commercial or financial relationships that could be constructed as a potential conflict of interest.

## Glossary

ARDS: acute respiratory distress syndrome
CGD: chronic granulomatous disease
CQ: chloroquine
COVID-19: coronavirus disease 2019
DMVs: double-membrane vesicles
HCQ: hydroxychloroquine
MHV: mouse hepatitis virus
RA: rheumatoid arthritis
SARS-CoV-2: severe acute respiratory syndrome Coronavirus 2
AOSD: Still disease
ACE-2: angiotensin-converting enzyme 2
AOSD: adult onset Still disease
CQ: chloroquine
DPP4: Human dipeptidyl peptidase 4
HCQ: hydroxychloroquine
IL: interleukin
TNF: tumor necrosis factor
WHO: World Health Organization
